# Woman*Response to a toast “To Woman,” Alumni (of Texas University) Association meeting, Amarillo, Texas, May 10, 1911.

**Published:** 1911-12

**Authors:** A. W. Fly

**Affiliations:** State Board of Health, Galveston, Texas


For Texas Medical Journal.

### Woman.*

BY A. W. FLY, M. D., STATE BOARD OF HEALTH, GALVESTON, TEXAS.

The subject upon which I have undertaken to address you is "Woman"—her influence on the progress of the world. This is undoubtedly one of the most interesting questions that could be submitted to any audience. Indeed, it is not only interesting, it is also very important. When we see how knowledge has civilized mankind; when we see how every great step, in the march and advance of nations, has been invariably preceded by a corresponding step in their knowledge; when we, moreover, see what is assuredly true, that women are constantly growing more influential, it becomes a matter of great moment that we should endeavor to ascertain the relation between their influence and our progress.

On every side, in all social phenomena, in the education of children, in the tone and spirit of literature, in the forms and usages of life; even in the proceedings of Legislatures, in the history of statute books, in the decisions of courts, we find manifold proof that women are gradually making their way, and slowly but surely winning for themselves a position superior to any they have attained in any age and in any nation of history. This is one of the many peculiarities which distinguish modern civilization, and which show how essentially the most advanced countries are different from those that formerly flourished. Among the most celebrated nations of antiquity, women held a very subordinate place. We have only to open the literature of the ancient Greeks to see with what airs of superiority, with what serene and lofty contempt, and sometimes with mocking and biting scorn women were treated by that lively and ingenious people. Instead of valuing them as companions, they looked on them as toys. How little part women really took in the development of Greek civilization may be illustrated by the singular fact that their influence, scanty as it was, did not reach its height in the most civilized times, or in the most civilized regions. In Sparta, women possessed more influence than they did in Athens; although the Spartans were rude and ignorant, the Athenians polite and accomplished. The causes of these inconsistencies would form a curious subject for investigation, but it is enough to call your attention

*Response to a toast "To Woman," Alumni (of Texas University) Association meeting, Amarillo, Texas, May 10, 1911.

to them as one of the many proofs that the boasted civilizations of antiquity were eminently one-sided, and that they fell because society did not advance in all its parts, but sacrificed the rights of women, in order to secure the progress of men.

In modern society, we have, happily, no instances of this kind; and if we inquire what influence of women has been upon that society, every one will concede that it has been extremely beneficial. Their influence has prevented life from being too exclusively practical and selfish, and has saved it from degenerating into a dull, monotonous routine, by infusing into it an ideal and romantic element. It has softened the violence of men; it has improved their manners; it has lessened their cruelty. I believe that this influence of women acts by virtue of certain laws inherent to human nature, and that, although it works as an undercurrent below the surface, it is, therefore, invisible to hasty observers. It has produced the most important results, and has affected the shape, the character and the amount of our knowledge.

Woman is, by nature, imaginative, while man strives to arrive at cold, barren facts, without using his imaginative powers. Now, there are several reasons why women prefer the imaginative, or, I may say, the ideal method. They are more emotional, more enthusiastic, and more trusting than men. They, therefore, live more in the ideal world, while men, with their colder, harder and more austere organizations, are more practical and more under the dominion of facts to which they, consequently, ascribe a higher importance. Another circumstance that makes women more imaginative is that they possess more of what is called intuition. They can not see so far as men can, but, what they do see, they see quicker. Hence, they are constantly tempted to grasp at once at an idea, and seek to solve a problem suddenly, in contrast to the slower and more laborious ascent of man.

From this point of view, you will see the incalculable service women have rendered to the progress of knowledge. Great and exclusive as is our passion for induction, it would, but for them, have been greater still. Empirical as we are, slaves as we are to the tyranny of facts, our slavery would, but for them, have been more complete and more ignominious. Their turn of thought, their habits of mind, their conversation, their influence, insensibly extending over the whole surface of society, and penetrating its intimate structure, have, more than all things put together, tended to raise us into an ideal world, lift us from the dust in which we are prone to grovel, and develop in us those germs of imagination which even the most sluggish and apathetic understandings in some degree possess. The striking fact that most men of genius have had remarkable mothers, and that they have gained from their mothers far more than from their fathers,—this singular and unquestionable fact can, I think, be best explained by those who understand the hopes and fond dreams of a loving mother.

We have every reason to believe that when the human mind once combines the whole of its power, when the imagination of woman, and the reasoning of man, are properly blended, we will be more than equal to the difficulties presented by the external world. As we surpass our fathers, so will our children surpass us. We, waging the forces of nature, what has too often been a precarious, unsteady and unskilled warfare, have never put forth the whole of our strength, because we have never given women full credit for her worth. When once we learn this great lesson, we will advance from success to success,—every struggle will issue in a conquest and every battle end in victory.

